# Cardiac Resynchronization Therapy Outcomes in Type 2 Diabetic Patients: Role of MicroRNA Changes

**DOI:** 10.1155/2016/7292564

**Published:** 2015-11-09

**Authors:** Celestino Sardu, Michelangela Barbieri, Maria Rosaria Rizzo, Pasquale Paolisso, Giuseppe Paolisso, Raffaele Marfella

**Affiliations:** Department of Medical, Surgical, Neurological, Metabolic and Aging Sciences, Second University of Naples, 80138 Naples, Italy

## Abstract

Heart failure (HF) and type 2 diabetes mellitus (T2DM) are two growing and related diseases in general population and particularly in elderly people. In selected patients affected by HF and severe dysfunction of left ventricle ejection fraction (LVEF), with left bundle brunch block, the cardiac resynchronization therapy with a defibrillator (CRT) is the treatment of choice to improve symptoms, NYHA class, and quality of life. CRT effects are related to alterations in genes and microRNAs (miRs) expression, which regulate cardiac processes involved in cardiac apoptosis, cardiac fibrosis, cardiac hypertrophy and angiogenesis, and membrane channel ionic currents. Different studies have shown a different prognosis in T2DM patients and T2DM elderly patients treated by CRT-D. We reviewed the literature data on CRT-D effect on adult and elderly patients with T2DM as compared with nondiabetic patients.

## 1. Introduction

Heart failure (HF) and type 2 diabetes mellitus (T2DM) are two related and growing health care problems [1, 2], with an increasing trend in elderly people [3]. The prevalence of T2DM in patients with HF is variable, ranging from 15% to 45% [4, 5], and T2DM is an independent risk factor for the development of HF [6], and it is associated with increased mortality in patients with HF [7].

In patients under optimal medical therapy with moderate to severe HF and cardiac dyssynchrony, cardiac resynchronization therapy (CRT) with a defibrillator improves contractile function and reverse ventricular remodeling, ameliorating symptoms, quality of life (QoL), and clinical outcomes [7, 8]. The CRT-induced “reverse molecular remodeling” in responders patients is related to an increase in the expression of genes involved in the regulation of excitation-contraction coupling and a reversal in the isoforms switching of the contractile genes [9] ultimately leading to remodeling effects and improving of myocardial performance. The effects in terms of reverse remodeling have been recently investigated in the REVERSE trial [10]. The effect of reverse remodeling was evaluated on long-term survival in mildly symptomatic patients with heart failure submitted to CRT [10]. In this trial authors [10] have reported a 68% reduction in mortality in patients with ≥15% decrease in left ventricle ejection systolic volume indexed as compared to the rest of the patients (*P* = 0.0004) and that the change in left ventricle ejection systolic volume indexed was a strong independent predictor (*P* = 0.0002) of long-term survival in mild HF. Therefore, we reviewed the literature data on CRT effect on adult and elderly patients with T2DM as compared with nondiabetic patients.

## 2. CRT and T2DM

In literature the most relevant studies and clinical trials on CRT-D clinical outcomes regard enrolled patients with mean age under 70 years. In fact, in the Multicenter Automatic Defibrillator Implantation Trial with CRT (MADIT-CRT) and the Cardiac Resynchronization-HF (CARE-HF), the mean age of enrolled patients was 65 and 66.4, respectively [11, 12]. Regarding the benefit of CRT in elderly people, authors have reported contrasting and different data [13–18].

On other hand in diabetic patients authors [22] have found a twofold higher mortality risk compared with nondiabetic patients; in particular, diabetic patients treated with insulin had a threefold higher mortality risk compared to nondiabetic patients, while diabetic patients not treated with insulin had only a modestly and nonsignificantly higher mortality risk (HR 1.59) compared to nondiabetic patients with HF [22]. Diabetes may affect patient prognosis after receiving a CRT-D (or a dual-chamber internal cardioverter defibrillator device), and authors have investigated this relevant aspect in the MADIT-RIT trial [23]. In this trial [23] the role of diabetes mellitus has been analyzed as predictor of appropriate/inappropriate internal defibrillator therapy. Dividing the study population in three groups (conventional therapy, high rate therapy, and delayed therapy) with similar percentage of diabetes mellitus (166/510, 32.5%; 155/491, 32.4%; and 160/482, 33.2%, resp.), authors [23] have shown that T2DM was a baseline clinical characteristic associated with mortality influence of ICD therapy by different heart rate ranges on mortality (HR: 1.64, 1.02–2.65; *P* value: 0.043). In this trial, inappropriate shock energy was reduced in the high rate and delayed therapy groups by 77% (*P* = 0.01) and 54% (*P* = 0.03), respectively, and these two programming approaches reduced potentially dangerous inappropriate therapies and increased survival among patients with ICDs [23]. Recently focusing on elderly diabetic patients, authors [22] have investigated the CRT response in diabetic patients >75 years old compared to nondiabetic subjects >75 years old. In this study, CRT was able to improve ventricular geometry and functional capacity in both diabetic and nondiabetic patients and the CRT effect was comparable in both populations [22]. In subgroups analysis conducted in diabetic patients the insulin therapy may determine a different effect as compared with oral antidiabetic therapy [18, 19, 21–24]. In fact insulin-dependent T2DM patients have a worse functional recovery and a worse prognosis after CRT [18, 19, 21–24]. These observations are confirmed by other authors by a post hoc analysis of the CARE-HF trial [11]. These results may be explained because patients receiving insulin might present a higher cardiovascular risk compared to those not receiving insulin. In fact there are adverse effects of insulin on cardiovascular function with an alteration in the relationship between the mitogenic and metabolic pathways leading to a worse cardiovascular disease pathogenesis progression [19] (Table 1).

## 3. MicroRNA

### 3.1. MicroRNA

MicroRNAs (miRs) first discovered in Caenorhabditis elegans are small (~22 nucleotides) noncoding RNAs [32]. miRs are transcribed by RNA polymerase II in a single-stranded nonprotein-coding RNAs defined intergenic miRs, often encoding various miRs, or generated by the processing of introns of protein-coding genes and defined intragenic or intronic miRs [33]. miRs are transcribed from multiple copies of genes that are located in the chromosomes as an integral part of the complex genome [34]. In a first step operated by RNA polymerase there is the generation of primary miRs (pri-miRs) with a stem-loop structure through transcription of intergenic miR genes II [35]. In a second step the pri-miR is processed to precursor miRs (pre-miRs) by the nuclear RNase endonuclease III, Drosha, and its partner proteins in the nucleus [36]. After this the pre-miRs are exported to the cytoplasm from the nucleus through the Ran-GTP-dependent nuclear pores exportin-5 [37] and in the cytoplasm, pre-miRs are further processed by another RNase III, Dicer, to become 22 nucleotide duplexes of mature miRs [35]. miRs may inhibit translation and/or promote mRNA degradation by base pairing to complementary sequences within the 3-untranslated region (3-UTR) of protein-coding mRNA transcripts [35, 36]. For this, miRs are implicated in epigenetic regulation of numerous cellular processes as proliferation, differentiation, and tumorigenesis [38], in a cascade way inducing hypo or hyper target genes expression [35, 39]. As suggested by authors [25, 26] miRs may be implicated in adaptive processes during heart failure disease and are for this reason evaluated as biomarkers.

### 3.2. Role of CRT on miRs Expression

In failing heart numerous adaptive processes may lead to the end stage of irreversible disease, not responsible for pharmacological therapeutic treatments, with an increased hospitalization and mortality trend [7]. In selected patients CRT improves contractile function inducing reverse ventricular remodeling and ameliorating symptoms, quality of life (QoL), and clinical outcomes [7, 8]. This clinical beneficial effect CRT is related to the manifestation of an improved myocardial performance, and reduction of NYHA functional class in responders patients [7, 8, 25, 26]. CRT may induce reversion of these adaptive processes, regulating cardiac apoptosis, fibrosis, and angiogenesis by direct genetic and epigenetic talking [9, 25, 26]. CRT-induced remodeling processes are related to alterations in genes and microRNA expression regulating cardiac apoptosis, cardiac fibrosis, cardiac hypertrophy, and angiogenesis [9, 25, 26]. These epigenetic effects are related to the modulation of 24 circulating miR patterns implicated in different processes of the failing heart in general population [25, 26]. These miRs are related to these cardiac adaptive processes and differently expressed after CRT [25, 26].

### 3.3. CRT Responders, miRs Expression, and Outcomes

In responders patients CRT may induce “reverse molecular remodeling,” which is related to an increased expression of genes involved in the regulation of excitation-contraction coupling and a reversal in the isoforms switching of the contractile genes [9] ultimately leading to remodeling effects and improving of myocardial performance. CRT effect as described [25, 26] may also regulate miRs expression. The improved myocardial performance in CRT responders is correlated with a significant increase in miR-26b-5p, miR-145-5p, miR-92a-3p, miR-30e-5p, and miR-29a-3p levels [25]. These miRs are involved in cardiac angiogenesis (miR-30, miR92, and miR-145), cardiac apoptosis (miR-30), cardiac fibrosis (miR-29), and membrane channel ionic currents (miR-26) and are mirrored by a significant reduction in BNP levels and might be involved in the process of cardiac functional recovery [25, 26] (Table 2). These adaptive processes have been differently investigated in failing heart and have shown a CRT-induced dynamic regulation [25, 26]. miRs are implied in dynamic regulative processes during heart failure stages and are so proposed as biomarkers differently expressed in CRT responders as compared with nonresponders patients [25, 26]. Therefore miRs are related to CRT-induced regulation of different cardiac adaptive processes and may identify and differentiate responders patients from nonresponders patients to CRT [25, 26]. On the other hand at baseline miRs are not identificative or predictive of CRT response [25]. These data have been evaluated in general population, while no data are present at the moment in diabetic patients and elderly diabetic patients. In these patients more studies have to be conducted regarding the CRT-induced epigenetic effect.

### 3.4. miR-26

miR-26 is a regulator of cellular proliferation, migration, invasion, and apoptosis in neoplastic diseases [40]. In cardiovascular disease, miR-26 is implicated in the control of critical signaling pathways relevant to endothelial cell growth, angiogenesis, and LV function after myocardial infarction [41]. miR-26 works by targeting the gene encoding inward-rectifier potassium current and it is downregulated in atrial samples from AF animals and patients [27]. In fact knockdown of endogenous miR-26 promoted AF in mice, whereas adenovirus-mediated expression of miR-26 reduced AF vulnerability [27]. miR-26 is a regulator of fibroblast cells functions and tissue fibrosis in congestive heart failure, by upregulation of fibroblast potassium channels expression and currents, with an increasing Ca^2+^ entry, and enhancing atrial fibroblast proliferation, promoting fibroblast remodeling and structural/arrhythmic consequences [42]. This miR, implicated in cardiac fibrotic adaptive processes as described before, is a selective miR target overexpressed by artificial electrical pacing during CRT in responders as compared to nonresponders patients [25, 26].

### 3.5. miR-29

miR-29 has been investigated as an oncomiR, with control effects on endometrial cell proliferation, apoptosis, and invasion [43]. In the setting of cardiovascular adaptive processes, miR-29 is a circulating miR implicated in regulation of myocardial fibrosis [44], targeting mRNAs that encode proteins involved in fibrosis as collagens, fibrillins, and elastin [28]. In myocardial infarction the downregulation of miR-29 with anti-miRs in vitro and in vivo induces the expression of collagens, whereas overexpression of miR-29 in fibroblasts reduces collagen expression, showing miR-29 as a miR regulator target of cardiac fibrosis [28]. The prominent controlling miR-29 effect on cardiac fibrosis is one adaptive mechanism of failing heart and it may be regulated by CRT [25, 26]. In fact in CRT responders miR-29 is significantly overexpressed, differentiating responders from nonresponders patients, and it is related to clinical improvement, associated with a reduction of NYHA class and BNP ematic levels [25, 26].

### 3.6. miR-30

miR-30 may perform numerous cellular control functions, as the regulation of adipocytes and osteoblasts differentiation [45]. miR-30 has been proposed as a biomarker in breast cancer by inhibiting cell migration and invasion [46]. In cardiovascular diseases miR-30 may regulate myocardial hypertrophy [47] and it has been studied as an implicated miR in acute myocardial infarction [48]. In a murine myocardial infarction model the overexpression of miR-30 family decreased cystathionine-*γ*-lyase expression, reduced hydrogen sulfide production, and then aggravated hypoxic cardiomyocyte injury [29]. Moreover, the therapeutical miR-30 inhibition has been proposed as a therapeutical target for myocardial ischemic disease [29]. miR-30 may control numerous cellular functions and it is implicated in cardiac angiogenesis and cardiac apoptosis and it is a significantly overexpressed miR during CRT treatment in responders patients [25, 26].

### 3.7. miR-92

miR-92 is an oncomiR implicated in leukemic disease [49] and breast cancer [50]. Recently authors have shown that oxidative stress may induce the expression of miR-92 in cultured endothelial cells and that circulating miR-92 level is inversely correlated with endothelial cell-dependent, flow-mediated vasodilation [30]. Moreover, in mice models, locked nucleic acid-modified antisense miR-92 attenuates inflammasome, improves vasodilation, and ameliorates angiotensin II-induced and aging-related atherogenesis [30]. This miR may control numerous cardiovascular effects mediated by oxidative stress, effects that may promote vascular tone (vascular dilation v/s constriction) and angiogenesis.

miR-92 is significantly overexpressed in CRT responders patients [25, 26], supposing in this way a regulative effect on neoangiogenesis in failing heart responder to CRT.

### 3.8. miR-145

miR-145 has been recently investigated as oncomiR in different cell type cancers as cervical cancer [51], human ovarian cancer [52], and pancreatic cancer [53], controlling cell proliferation, invasion, and migration. miR-145 expression is regulated by mechanical stretch in vascular smooth muscle cell phenotype [54] and it has shown a smooth muscle cell phenotype regulative effect [55]. miR-145 is a target miR for angiogenesis and in rabbit models the genetically engineered miR-145 (smart miR-145), restoring the downregulated miR-145 (in proliferative rat vascular smooth muscle cells and in rat carotid arteries with balloon injury and mouse atherosclerotic aortas), has shown therapeutic effects on the abnormal growth of vascular smooth muscle cells [31]. This target miR is induced and overexpressed during reverse remodelling processes in CRT responders [25, 26].

## 4. Conclusion

CRT may lead to left ventricular reverse remodeling, with LVEF, NYHA functional class, and 6MWT improvement in both diabetic and nondiabetic adult patients [22].

**Figure 1:**
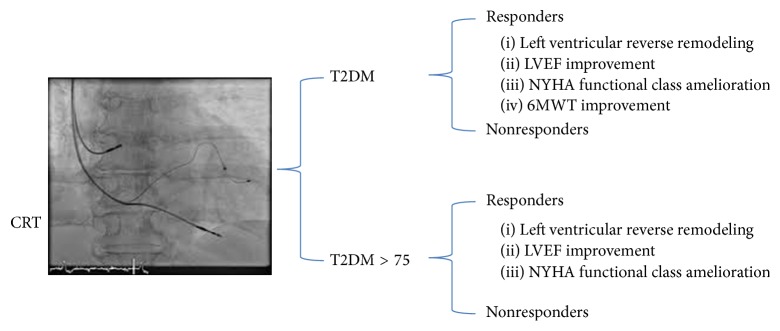
In this figure, the representation of CRT (cardiac resynchronization therapy) effect on T2DM (type 2 diabetes mellitus) and T2DM > 75 (type 2 diabetes mellitus older than 75 years) patients. In radioscopic image three catheters were placed in right atrial appendage (10 o'clock), right ventricular apex (5 o'clock), and coronary sinus lateral vein (3 o'clock). This is an example of biventricular pacing for cardiac resynchronization therapy. In responders there is a left ventricular reverse remodeling, a left ventricle ejection fraction (LVEF) improvement, an amelioration in New York Heart Association (NYHA) functional class, and 6 minutes walking test (6MWT) improvement. This last parameter (6MWT) is not improved in T2DM > 75.

These observations have been confirmed by other authors [24] in a population of elderly diabetic patients, a part of 6MWT improvement, that is not CRT-induced in elderly diabetes (Figure 1).

The lack of efficacy on 6MWT may be related to the degenerative effect of the aging on locomotor system and by multifactorial risk factors present in elderly patients [24]. T2DM patients treated by CRT have a higher total mortality than nondiabetic patients, independent of baseline characteristics [22]. The worse prognosis in CRT-treated T2DM may be due mainly to the higher mortality of patients with insulin-treated diabetes [19, 21–24]. On the other hand observing the CRT-D effect in T2DM population at-risk cardiac patients, authors have evidenced a substantial reduction in the risk of HF or death and a significative improvement in cardiac remodeling in those with ischemic and nonischemic cardiomyopathy, with a more pronounced benefit in patients with nonischemic disease [20]. From these observations T2DM, regardless of the therapy used to treat it and the presence of coronary artery disease, did not influence the beneficial effect of CRT on any end point [20, 21]. Focusing on the CRT-D response in a population of elderly T2DM patients (patients >75 years old) and particularly on the potential functional role of T2DM regarding the effectiveness of CRT, authors [24] have demonstrated that diabetic patients >75 years old exhibit a response to CRT that is comparable to nondiabetic subjects. From these observations T2DM patients at every age may exhibit a clinical CRT response that is not different as observed in nondiabetic patients. T2DM patients with HF need to be treated with CRT, following the same clinical and instrumental indications as reported for general population. Regarding the different prognosis observed in T2DM treated by insulin, we could conclude that the insulin therapy may identify a different population of diabetic patients with multifactorial risk factors and an advanced heart disease. The pathological mechanisms present in the insulin T2DM patients may affect in different way the prognosis as compared with noninsulin dependent diabetic patients. From this observation a differential analysis of the impact of the various oral hypoglycemic agents or the different insulin preparations needs to be performed in next clinical studies. Another point is represented by the epigenetic effect in T2DM patients treated by CRT, which has never been investigated until now. The CRT epigenetic effect in T2DM population may be related to miR expression patterns regulation, and miR expression may be used as a monitoring marker of CRT response in T2DM, to differentiate responders from nonresponders patients as suggested in general population [25]. The identification of CRT-induced miR expression pathways in T2DM represents an effect that has to be investigated in future research trials. Until now no data were reported regarding the epigenetic pathways regulated in these patients. In future, elderly and diabetic elderly will be studied to evaluate the impact of CRT on miRs expression. Authors [25, 26] have proposed that in CRT responder patients there is a modulation of several miRs. These miRs are hypo- or hyperexpressed by CRT and they may be differentiating biomarkers between CRT responders as compared to CRT nonresponders patients. The mechanistic role of circulating miRs modulated after CRT [25, 26] has not been fully investigated and it still needs to be investigated in future researches. For all these reasons the circulating miR levels do not always reflect changes in the intracellular setting. Therefore, these results should not lead to conclusions regarding the benefit of CRT, or the lack of the CRT-related benefit, in diabetic and elderly patients and its modulative effect on miR's changing expression and it has to be investigated in future research trials.

## 5. Future Purpose

We can suppose that particular population as elderly and diabetic patients may present a more complex and different disease stages with regard to aging and diabetes as compared to general population, with more pronounced fibrotic deposition, apoptotic myocardial cells deaths, and loss of cellular repair functions and angiogenesis. The translational approach as reported for general population [25, 26] may be applied also in these future researches fields, with particular attention to epigenetic regulation of cardiac apoptosis, cardiac fibrosis, and angiogenesis. The identification of these pathways may introduce the opportunity to describe new data and take care, at best management, of these particular populations. As described for general population [25, 26], new researches and pharmacological therapies may lead in the future to envelopment of selected and specific miR enhancing (adenovirus) or blocking agents (antagomiR) to improve clinical outcomes also in nonresponders T2DM patients. New therapeutical options for modulating miRs expression (hypo- and/or hyperexpression) in vivo have been discussed recently by authors [56]. These authors have proposed a pharmacological modulation of miRs activity by different approaches [56]. Synthetic miR mimics may restore the function of a target miR, and a conjugation strategy with the nucleic acid linked to targeting molecules, such as peptides, antibodies, or other bioactive molecules, may promote homing of the miR modulator to specific cell types [56]. Moreover the antimiR or miR mimic could be encapsulated into a lipid-based formulation that enhances cell-specific uptake [56]. Another approach to target directly the delivery issue may be represented by device-based delivery approaches, such as stents or catheters, local injections, or ectopical delivery [56]. Similarly, development of miR replacement therapies will require optimization for restoring the activity of a downregulated or lost miR, while preventing the introduction of supraphysiological levels of the same miR [56]. In a recent trial [57] authors have described the use of miravirsen, a locked nucleic 12 acid-modified antisense oligonucleotide, in patients with chronic hepatitis C virus genotype 1 infection. The miravirsen sequesters mature miR-122 in a highly stable heteroduplex, leading to the functional inhibition of miR-122. In this clinical phase 2 authors study showed that the antimiR-122 drug miravirsen was safe and well-tolerated. Moreover in this population the use of miravirsen has showed prolonged dose-dependent reductions in HCV RNA levels without evidence of viral resistance [57]. According to these observations other authors [58] have recently proposed a microRNA-based therapeutical approach to inhibit vascular smooth cells proliferation in vitro and in vivo models. Using an adenoviral vector that encodes cyclin-dependent kinase inhibitor p27 with target sequences for EC-specific miR-126-3p at the 3′ end, authors [58] have shown in treated animals an overexpression of exogenous p27 in VSMCs, demonstrating the potential of using a miR-based strategy as a therapeutic approach to specifically inhibit vascular restenosis while preserving EC function. Taken all together, these experimental and innovative results are teaching us that we can start to use miR selective therapy. The time to translate these innovations in clinical practice is now, and we have to think more about new therapeutical approach to treat (by miR target therapy) non-CRT responders humans.

## Figures and Tables

**Table 1:** In this table, from left to right, the following columns are reported: the number of patients, mean age of study population (in case of more than one group of patients, more mean ages are reported and compared), diabetes (diabetic diseases, yes or no), D. patients (number of diabetic patients), insulin therapy (insulin therapy, yes or no), and references, in alphabetic order with first name of authors and assigned numbers. The symbol — is used in case of information not found in the reference studies.

Number of patients	Mean age	Diabetes	D. patients	Insulin therapy	References
170	59 ± 9 versus 76 ± 4	—	—	—	Bleeker et al. [13]
813	66.4	Yes	207	Yes	Cleland et al. (CARE-HF trial) [12]
355	64 ± 7 versus 62 ± 10	Yes	141	Yes	Fantoni et al. [19]
330	83.7 ± 2.6 versus 66.9 ± 9.5	—	—	—	Foley et al. [16]
1787	<65 versus 65–74 versus ≥75	Yes	26 (5%) versus 55 versus (7%) 27 (6%)	Yes	Fumagalli et al. [15]
552	67 ± 9 versus 63 ± 10	Yes	552	Yes	George et al. [20]
813	—	Yes	207	Yes	Hoppe et al. [21]
447	65.4 ± 10.2 versus 66.6 ± 7.5	Yes	91	Yes	Mangiavacchi et al. [22]
1820	65	Yes	552	Yes	Martin et al. (MADIT-CRT) [11]
1500	66 ± 14	Yes	481	—	Ruwald et al. (MADIT-RIT) [23]
610	61.8 ± 11.6 versus 62.9 ± 10.6	Yes	46 versus 91	—	Gold et al. (REVERSE) [10]
3327	<70 versus >70	—	—	—	Pulignano et al. [14]
72	81.4 ± 5.8 versus 82.5 ± 6.9	Yes	32	Yes	Sardu et al. [24]

**Table 2:** In this table, the representation of microRNA (miR), cardiac resynchronization therapy (CRT) effect on miRs expression, the miR's epigenetic effect, and the potential role in heart remodelling is displayed. On the right column, the bibliography reference for every investigated miR is represented. IK1 is inward-rectifier K^+^ current; SREBP2 is sterol regulatory element-binding protein 2 (an oxidative stress-induced protein); PI3 is phosphoinositide 3-kinase; Akt is serine/threonine kinase; p53 is p53 protein.

Investigated miRNA	CRT effect	Epigenetic effect	Potential role in heart remodelling	Bibliography
*miR-26b-5p *	Significant increase	IK1 and Ca^2+^ current	Membrane channel ionic currents	[25–27]
*miR-29a-3p *	Significant increase	Collagen, fibrillin, and elastin	Cardiac fibrosis	[25, 26, 28]
*miR-30e-5p *	Significant increase	Angiotensin II, cystathionine-*γ*-lyase (CyL), and hydrogen sulfide (HS)	Cardiac angiogenesis and cardiac apoptosis	[25, 26, 29]
*miR-92a-3p *	Significant increase	SREBP2 and angiotensin II	Cardiac angiogenesis	[25, 26, 30]
*miR-145-5p *	Significant increase	PI3 Kinase/Akt/p53	Cardiac angiogenesis	[25, 26, 31]

## References

[1] Eurich D. T., Weir D. L., Majumdar S. R. (2013). Comparative safety and effectiveness of metformin in patients with diabetes mellitus and heart failure: systematic review of observational studies involving 34,000 patients. *Circulation: Heart Failure*.

[2] Santulli G. (2013). Epidemiology of cardiovascular disease in the 21st century: updated numbers and updated facts. *Journal of Cardiovascular Disease*.

[3] Huang E. S., Laiteerapong N., Liu J. Y., John P. M., Moffet H. H., Karter A. J. (2014). Rates of complications and mortality in older patients with diabetes mellitus: the diabetes and aging study. *JAMA Internal Medicine*.

[4] Peterson L. R., McKenzie C. R., Schaffer J. E. (2012). Diabetic cardiovascular disease: getting to the heart of the matter. *Journal of Cardiovascular Translational Research*.

[5] Cubbon R. M., Adams B., Rajwani A. (2013). Diabetes mellitus is associated with adverse prognosis in chronic heart failure of ischemic and non ischemic etiology. *Diabetes & Vascular Disease Research*.

[6] Nodari S., Manerba A., Vaccari A. (2012). Six-year prognosis of diabetic patients with coronary artery disease. *European Journal of Clinical Investigation*.

[7] Moss A. J., Hall W. J., Cannom D. S. (2009). Cardiac-resynchronization therapy for the prevention of heart-failure events. *The New England Journal of Medicine*.

[8] Peterson P. N., Greiner M. A., Qualls L. G. (2013). QRS duration, bundle-branch block morphology, and outcomes among older patients with heart failure receiving cardiac resynchronization therapy. *The Journal of the American Medical Association*.

[9] Vanderheyden M., Mullens W., Delrue L. (2008). Myocardial gene expression in heart failure patients treated with cardiac resynchronization therapy. Responders versus non responders. *Journal of the American College of Cardiology*.

[10] Gold M. R., Daubert C., Abraham W. T. (2015). The effect of reverse remodeling on long-term survival in mildly symptomatic patients with heart failure receiving cardiac resynchronization therapy: results of the REVERSE study. *Heart Rhythm*.

[11] Martin D. T., McNitt S., Nesto R. W., Rutter M. K., Moss A. J. (2011). Cardiac resynchronization therapy reduces the risk of cardiac events in patients with diabetes enrolled in the multicenter automatic defibrillator implantation trial with cardiac resynchronization therapy (MADIT-CRT). *Circulation: Heart Failure*.

[12] Cleland J. G. F., Daubert J.-C., Erdmann E. (2005). The effect of cardiac resynchronization on morbidity and mortality in heart failure. *The New England Journal of Medicine*.

[13] Bleeker G. B., Schalij M. J., Molhoek S. G. (2005). Comparison of effectiveness of cardiac resynchronization therapy in patients <70 versus ≥70 years of age. *The American Journal of Cardiology*.

[14] Pulignano G., Del Sindaco D., Tavazzi L. (2002). Clinical features and outcomes of elderly outpatients with heart failure followed up in hospital cardiology units: data from a large nationwide cardiology database (IN-CHF registry). *The American Heart Journal*.

[15] Fumagalli S., Valsecchi S., Boriani G. (2011). Comparison of the usefulness of cardiac resynchronization therapy in three age-groups (<65, 65–74 and ≥75 Years) (from the InSync/InSync ICD Italian Registry). *The American Journal of Cardiology*.

[16] Foley P. W. X., Chalil S., Khadjooi K., Smith R. E. A., Frenneaux M. P., Leyva F. (2008). Long-term effects of cardiac resynchronization therapy in octogenarians: a comparative study with a younger population. *Europace*.

[17] Cutro R., Rich M. W., Hauptman P. J. (2012). Device therapy in patients with heart failure and advanced age: too much too late?. *International Journal of Cardiology*.

[18] Kramer D. B., Reynolds M. R., Mitchell S. L. (2013). Resynchronization: considering device-based cardiac therapy in older adults. *Journal of the American Geriatrics Society*.

[19] Fantoni C., Regoli F., Ghanem A. (2008). Long-term outcome in diabetic heart failure patients treated with cardiac resynchronization therapy. *European Journal of Heart Failure*.

[20] George J., Barsheshet A., Moss A. J. (2012). Effectiveness of cardiac resynchronization therapy in diabetic patients with ischemic and nonischemic cardiomyopathy. *Annals of Noninvasive Electrocardiology*.

[21] Hoppe U. C., Freemantle N., Cleland J. G. F., Marijianowski M., Erdmann E. (2007). Effect of cardiac resynchronization on morbidity and mortality of diabetic patients with severe heart failure. *Diabetes Care*.

[22] Mangiavacchi M., Gasparini M., Genovese S. (2008). Insulin-treated type 2 diabetes is associated with a decreased survival in heart failure patients after cardiac resynchronization therapy. *Pacing and Clinical Electrophysiology*.

[23] Ruwald A. C., Schuger C., Moss A. J. (2014). Mortality reduction in relation to implantable cardioverter defibrillator programming in the Multicenter Automatic Defibrillator Implantation Trial-Reduce Inappropriate Therapy (MADIT-RIT). *Circulation: Arrhythmia and Electrophysiology*.

[24] Sardu C., Marfella R., Santulli G. (2014). Impact of diabetes mellitus on the clinical response to cardiac resynchronization therapy in elderly people. *Journal of Cardiovascular Translational Research*.

[25] Marfella R., Di Filippo C., Potenza N. (2013). Circulating microRNA changes in heart failure patients treated with cardiac resynchronization therapy: responders vs. non-responders. *European Journal of Heart Failure*.

[26] Sardu C., Marfella R., Santulli G., Paolisso G. (2014). Functional role of miRNA in cardiac resynchronization therapy. *Pharmacogenomics*.

[27] Luo X., Pan Z., Shan H. (2013). MicroRNA-26 governs profibrillatory inward-rectifier potassium current changes in atrial fibrillation. *The Journal of Clinical Investigation*.

[28] van Rooij E., Sutherland L. B., Thatcher J. E. (2008). Dysregulation of microRNAs after myocardial infarction reveals a role of miR-29 in cardiac fibrosis. *Proceedings of the National Academy of Sciences of the United States of America*.

[29] Shen Y., Shen Z., Miao L. (2015). miRNA-30 family inhibition protects against cardiac ischemic injury by regulating cystathionine-*γ*-lyase expression. *Antioxidants & Redox Signaling*.

[30] Chen Z., Wen L., Martin M. (2015). Oxidative stress activates endothelial innate immunity via sterol regulatory element binding protein 2 (SREBP2) transactivation of MicroRNA-92a. *Circulation*.

[31] Liu X., Cheng Y., Yang J. (2013). Flank sequences of miR-145/143 and their aberrant expression in vascular disease: mechanism and therapeutic application. *Journal of the American Heart Association*.

[32] Ruvkun G., Giusto J. (1989). The *Caenorhabditis elegans* heterochronic gene lin-14 encodes a nuclear protein that forms a temporal developmental switch. *Nature*.

[33] Ambros V., Bartel B., Bartel D. P. (2003). A uniform system for microRNA annotation. *RNA*.

[34] Bartel D. P., Chen C.-Z. (2004). Micromanagers of gene expression: the potentially widespread influence of metazoan microRNAs. *Nature Reviews Genetics*.

[35] Kim V. N. (2005). MicroRNA biogenesis: coordinated cropping and dicing. *Nature Reviews Molecular Cell Biology*.

[36] Bartel D. P. (2004). MicroRNAs: genomics, biogenesis, mechanism, and function. *Cell*.

[37] Lund E., Güttinger S., Calado A., Dahlberg J. E., Kutay U. (2004). Nuclear export of microRNA precursors. *Science*.

[38] Denli A. M., Tops B. B. J., Plasterk R. H. A., Ketting R. F., Hannon G. J. (2004). Processing of primary microRNAs by the Microprocessor complex. *Nature*.

[39] Van Rooij E., Olson E. N. (2012). MicroRNA therapeutics for cardiovascular disease: opportunities and obstacles. *Nature Reviews Drug Discovery*.

[40] Du J. Y., Wang L. F., Wang Q., Yu L. D. (2015). miR-26b inhibits proliferation, migration, invasion and apoptosis induction via the downregulation of 6-phosphofructo-2-kinase/fructose-2,6-bisphosphatase-3 driven glycolysis in osteosarcoma cells. *Oncology Reports*.

[41] Icli B., Dorbala P., Feinberg M. W. (2014). An emerging role for the miR-26 family in cardiovascular disease. *Trends in Cardiovascular Medicine*.

[42] Qi X. Y., Huang H., Ordog B. (2015). Fibroblast inward-rectifier potassium current upregulation in profibrillatory atrial remodeling. *Circulation Research*.

[43] Long M., Wan X., La X., Gong X., Cai X. (2015). miR-29c is downregulated in the ectopic endometrium and exerts its effects on endometrial cell proliferation, apoptosis and invasion by targeting c-Jun. *International Journal of Molecular Medicine*.

[44] Dai Y., Dai D., Mehta J. L. (2014). MicroRNA-29, a mysterious regulator in myocardial fibrosis and circulating miR-29a as a biomarker. *Journal of the American College of Cardiology*.

[45] Wang J., Guan X., Guo F. (2013). miR-30e reciprocally regulates the differentiation of adipocytes and osteoblasts by directly targeting low-density lipoprotein receptor-related protein 6. *Cell Death & Disease*.

[46] Cheng C. W., Wang H. W., Chang C. W. (2012). MicroRNA-30a inhibits cell migration and invasion by down regulating vimentin expression and is a potential prognostic marker in breast cancer. *Breast Cancer Research and Treatment*.

[47] Pan W., Zhong Y., Cheng C. (2013). MiR-30-regulated autophagy mediates angiotensin II-induced myocardial hypertrophy. *PLoS ONE*.

[48] Long G., Wang F., Duan Q. (2012). Circulating miR-30a, miR-195 and let-7b associated with acute myocardial infarction. *PLoS ONE*.

[49] Brockway S., Zeleznik-Le N. J. (2015). WEE1 is a validated target of the microRNA miR-17-92 cluster in leukemia. *Cancer Genetics*.

[50] Chacon-Cortes D., Smith R. A., Lea R. A., Youl P. H., Griffiths L. R. (2015). Association of microRNA 17–92 cluster host gene (MIR17HG) polymorphisms with breast cancer. *Tumor Biology*.

[51] Ye C., Sun N. X., Ma Y. (2015). MicroRNA-145 contributes to enhancing radiosensitivity of cervical cancer cells. *FEBS Letters*.

[52] Liang H., Jiang Z., Xie G., Lu Y. (2015). Serum microRNA-145 as a novel biomarker in human ovarian cancer. *Tumor Biology*.

[53] Han T., Yi X. P., Liu B., Ke M. J., Li Y. X. (2015). MicroRNA-145 suppresses cell proliferation, invasion and migration in pancreatic cancer cells by targeting NEDD9. *Molecular Medicine Reports*.

[54] Hu B., Song J. T., Qu H. Y. (2014). Mechanical stretch suppresses microRNA-145 expression by activating extracellular signal-regulated kinase 1/2 and upregulating angiotensin-converting enzyme to alter vascular smooth muscle cell phenotype. *PLoS ONE*.

[55] Ohnaka M., Marui A., Yamahara K. (2014). Effect of microRNA-145 to prevent vein graft disease in rabbits by regulation of smooth muscle cell phenotype. *The Journal of Thoracic and Cardiovascular Surgery*.

[56] van Rooij E., Kauppinen S. (2014). Development of microRNA therapeutics is coming of age. *EMBO Molecular Medicine*.

[57] Janssen H. L. A., Reesink H. W., Lawitz E. J. (2013). Treatment of HCV infection by targeting microRNA. *The New England Journal of Medicine*.

[58] Santulli G., Wronska A., Uryu K. (2014). A selective microRNA-based strategy inhibits restenosis while preserving endothelial function. *The Journal of Clinical Investigation*.

